# Protein kinase C epsilon activation regulates proliferation, migration, and epithelial to mesenchymal-like transition in rat Schwann cells

**DOI:** 10.3389/fncel.2023.1237479

**Published:** 2023-08-14

**Authors:** Tasnim Mohamed, Alessandra Colciago, Marina Montagnani Marelli, Roberta Manuela Moretti, Valerio Magnaghi

**Affiliations:** Department of Pharmacological and Biomolecular Sciences “Rodolfo Paoletti”, University of Milan, Milan, Italy

**Keywords:** DCP-LA, EMT, BDNF, myelin, cyclotraxin B

## Abstract

**Introduction:**

Protein kinase type C-ε (PKCε) plays an important role in the sensitization of primary afferent nociceptors, promoting mechanical hyperalgesia. In accordance, we showed that PKCε is present in sensory neurons of the peripheral nervous system (PNS), participating in the control of pain onset and chronification. Recently, it was found that PKCε is also implicated in the control of cell proliferation, promoting mitogenesis and metastatic invasion in some types of cancer. However, its role in the main glial cell of the PNS, the Schwann cells (SCs), was still not investigated.

**Methods:**

Rat primary SCs culture were treated with different pharmacologic approaches, including the PKCε agonist dicyclopropyl-linoleic acid (DCP-LA) 500 nM, the human recombinant brain derived neurotrophic factor (BDNF) 1 nM and the TrkB receptor antagonist cyclotraxin B 10 nM. The proliferation (by cell count), the migration (by scratch test and Boyden assay) as well as some markers of SCs differentiation and epithelial-mesenchymal transition (EMT) process (by qRT-PCR and western blot) were analyzed.

**Results:**

Overall, we found that PKCε is constitutively expressed in SCs, where it is likely involved in the switch from the proliferative toward the differentiated state. Indeed, we demonstrated that PKCε activation regulates SCs proliferation, increases their migration, and the expression of some markers (e.g., glycoprotein P0 and the transcription factor Krox20) of SCs differentiation. Through an autocrine mechanism, BDNF activates TrkB receptor, and controls SCs proliferation via PKCε. Importantly, PKCε activation likely promoted a partial EMT process in SCs.

**Discussion:**

PKCε mediates relevant actions in the neuronal and glial compartment of the PNS. In particular, we posit a novel function for PKCε in the transformation of SCs, assuming a role in the mechanisms controlling SCs' fate and plasticity.

## Introduction

The ε isoform of protein kinase C (PKCε) is a kind of Ca^++^-independent protein kinase widely expressed through the mammalian organism, which possesses substantial roles in the function of the immune and nervous systems (Aksoy et al., 2004; Shirai et al., 2008). Importantly, PKCε plays a role in primary afferent nociceptor sensitization and mechanical hyperalgesia (Khasar et al., 1999; Srinivasan et al., 2008; Villarreal et al., 2009). Indeed, PKCε has been shown to be crucial in establishing a long-lasting sensitization termed hyperalgesic priming (Ferrari et al., 2015). In primed animals, the long-lasting hyperalgesia is blocked by selective PKCε inhibition (Aley et al., 2000). More recently, it was demonstrated that PKCε exerts other remarkable actions, controlling cell proliferation and promoting metastasis in several types of cancer, such as breast cancer cells; this activity occurs through induction of the epithelial to mesenchymal transition (EMT) process (Pan et al., 2005; Gorin and Pan, 2009; Jain and Basu, 2014). EMT is a biological phenomenon by which epithelial cells lose their differentiated characteristics (i.e. cell adhesion and polarity) and acquire mesenchymal features, such as spindle-shaped morphology and increased migration (Savagner, 2010). It was observed, indeed, that the loss of epithelial hallmarks was coupled with the upregulation of several genes facilitating migration. In this light, EMT contributes to some physiopathologic processes such as wound healing, fibrosis, and cancer (Dongre and Weinberg, 2019). EMT can also occur in non-epithelial cells, or even, in cells with reduced epithelial properties, and is termed EMT-like (Iser et al., 2019). Therefore, the activation of an EMT program does not necessarily drive cells toward a full phenotype, but rather to hybrid states, such that some cells undergo the so-called partial EMT (Verstappe and Berx, 2023).

There is evidence that PKCε acts by phosphorylating some ion channels, including TRPV1 (Amadesi et al., 2006; Bogen et al., 2012), or decreasing the GABA_A_ receptor inhibitory effect (Qi et al., 2007) and increasing the neuronal excitability. Indeed, PKCε was found to be able to reduce the GABA_A_ receptor sensitivity to an allosteric modulator, the neuroactive steroid allopregnanolone (ALLO) (Hodge et al., 1999). In the peripheral nervous system (PNS), ALLO is synthesized and released by Schwann cells (SCs) (Faroni and Magnaghi, 2011; Bonalume et al., 2020; Colciago et al., 2020), the main glial cells forming the myelin sheath, and targets both neurons and glial cells. ALLO participates in the regulation of peripheral myelination, nerve regeneration (Faroni and Magnaghi, 2011; Melfi et al., 2017; Colciago et al., 2020), and nociception (Gonzalez et al., 2019; Bonalume et al., 2020).

Interestingly, recent evidence indicates that PKCε is present in both myelinated and unmyelinated fibers (i.e., primary afferent nociceptors) of the PNS, mostly localized in the axonal compartment, but also moderately present in SCs (Kawano et al., 1997). Thereby, a novel crosstalk between the SCs and peripheral sensory neurons in regulating the neuronal PKCε has been shown (Puia et al., 2015; Bonalume et al., 2020). SCs tonically release ALLO, which in turn, autocrinally, upregulated the synthesis and release of the brain-derived neurotrophic factor (BDNF). Then, the SCs-released BDNF activates PKCε in sensory neurons, *via* the TrkB receptor, probably participating in the control of pain onset and chronification (Bonalume et al., 2020).

Based on this evidence, the logical aim of this study was to investigate whether PKCε may have any direct biological role also in the SCs of the PNS. Our findings confirmed the presence of PKCε in the PNS and in rat primary SCs. We highlighted that PKCε takes part in the SCs switch from the proliferative toward the differentiated state; indeed, proliferation was decreased, while markers of SC differentiation were increased. Interestingly, PKCε activation likely promoted a partial EMT-like process in rat SCs, enhancing the migratory phenotype. These mechanisms involve an autocrine regulation of SCs through the BDNF/TrkB axis.

## Materials and methods

### Animals

All experiments involved newborn Sprague–Dawley rats (Charles River, Calco, Italy) and were performed in accordance with current European rules concerning the care and use of animals (Council Directive 2010/63/EU of the European Parliament and the Council of 22 September 2010 on the protection of animal used for scientific purposes) and 3R's guidelines. The Ethical Committee of the University of Milan approved the use of animals for cell culture.

### SCs primary cultures

SCs cultures were obtained as previously described (Magnaghi et al., 2006; Melfi et al., 2017). Rat sciatic nerves were digested with 1% collagenase and 0.25% trypsin (Merk Life Science, Milan, Italy), then mechanically dissociated, filtered through a 100-μm filter (BD Biosciences, Milan, Italy), and centrifuged for 5 min at 900 rpm. Pellets were suspended in Dulbecco's modified Eagle's medium (DMEM, Serotec, Oxford, UK) plus 10% fetal calf serum (FCS; Thermo Fisher Scientific, Waltham, MA, USA) and plated on 35 mm Petri dishes. After 24 h, the medium was supplemented with 10 μM Ara-C (Merk Life Science). The medium was then changed with DMEM-FCS 10% plus 10 μM forskolin (Merk Life Science) and 200 μg/ml bovine pituitary extract (BPE; Thermo Fisher Scientific). Cells became confluent in 10 days. Immunopanning for final purification was carried out by incubating the cells for 30 min with mouse anti-rat Thy1.1 antibody (Bio-Rad Laboratories, Segrate, Italy), followed by 500 μl of baby rabbit complement (Cedarlane, Burlington, Canada). Cell suspension (6 × 10^4^ cells) was seeded on 35 mm Petri dishes, in the presence of 2 μM forskolin. At the third *in vitro* passage, SCs were treated for 48 h with 4 μM forskolin and then used for different assays. SCs purity (more than 98%) was tested with a specific antibody against glycoprotein P0 (Mauro et al., 2013).

### Pharmacological treatments

The desired concentration of each substance was achieved by the dilution of stock into the culture medium. Substances used were dicyclopropyl-linoleic acid (DCP-LA; Merck Life Science) 500 nM, selective PKCε activator; cyclotraxin B 10 nM (Tocris, Bio-Techne, Milan, Italy); and human recombinant BDNF 1 nM (Merk Life Since). Drug concentration was set in previous experiments (Kawano et al., 1997; Bonalume et al., 2020), without toxic effects. Control cultures were treated with vehicle (DMSO). Differentiated SCs primary cultures were treated for the indicated times after overnight serum-free medium exposure. Medium changes and pharmacological treatments were done every other day.

### qRT-PCR

RNA samples from SCs cultures were extracted using TRIzol^TM^ (Thermo Fisher) according to the manufacturer's protocol and quantified with NanoDrop2000 (Thermo Fisher). Pure RNA was obtained after DNAse treatment with a specific kit (Merk Life Science); 1 μg of RNA was reverse-transcribed to cDNA using iScript™ Reverse Transcription Supermix for RT-qPCR (Bio-Rad Laboratories). Primers were designed by the PrimerBlast software (NIH, Bethesda, MD, USA); primer sequences are presented in Table 1. The primer efficiencies were experimentally set up for each couple of primers. An amount of 10 ng of cDNA for each sample was used for real-time PCR. qRT-PCR was performed by measuring the incorporation of SsoFast^TM^ EvaGreen^®^ dye (Bio-Rad Laboratories) with a CFX 96 Real Time System-C1000 touch thermal cycler (Bio-Rad). Data analysis was performed using the CFX Manager 2.0 software (Bio-Rad Laboratories). The threshold cycle number (Ct) values of both the calibrator and the samples of interest were normalized to the geometric mean of Ct of the endogenous housekeeping genes. Data analysis was performed with the comparative threshold cycle and results are expressed as 2^−ΔΔCt^. RNA obtained from control samples was used as a reference.

**Table 1:** Sequences of primers used in the qRT-PCR.

**Primer name**	**Forward primer 5'−3'**	**Reverse primer 5'−3'**
PKCε	CCCCTTGTGACCAGGAACTA	AGCTGGCCATCAGTAGACGA
MAG	AATGCCTATGGCCAGGACAACC	TGTGGGCTTCCAAGGTGCATAC
P0	CCTGCTCTTCTCTTCTTTG	CACAGCACCATAGACTTC
PMP22	TCCTGTTCCTTCACATCG	TGCCAGAGATCAGTCCTG
c-Jun	TGCCTCCAAGTGCCGGAAAA	CAGCTCGGAGTTTTGCGCTT
Sox10	ACGCAGAAAGTTAGCCGACCA	TCACTCTCGTTCAGCAACCTCCA
Krox20	CTCTCAGTGGTTTTATGCACCAGC	TCATGCCATCTCCAGCCACT
Shh	GCTGGATTCGACTGGGTCTACTAT	CCACGGAGTTCTCTGCTTTCAC
α-tubulin	TCGCGCTGTAAGAAGCAACACC	GGAGATACACTCACGCATGGTTGC
18S	CTGCCCTATCAACTTTCGATGGTAG	CCGTTTCTCAGGCTCCCTCTC

### Immunofluorescence

Nerves were explanted and desheeted, then fixed in 4% paraformaldehyde (PFA, Merk Life Science), included in OCT (Sakura Finetek, Torrance, CA, USA), and cut in cross sections. For longitudinal teased fibers, slight digestion was performed by incubating nerve fragments in collagenase IV (Merk Life Science) for 45 min, before fixing them in 4% PFA, and then including them in OCT. Cells were plated on coverslips, fixed in 4% PFA, and processed for immunostaining. Primary antibodies were the following: rabbit anti-S100 1:150 (DAKO Agilent, Santa Clara, CA, USA), rabbit anti-PKCε 1:200 (Abcam, Cambridge, UK), rabbit anti-phospho S729 PKCε 1:200 (Abcam), and mouse anti-SMI31/32 1:500 (Biolegend, San Diego, CA, USA). To reveal SC, cytoskeleton was used, and phalloidin-FITC 1:250 (Merk Life Science) was used to stain f-actin. After washing, slides were mounted using Vectashield^TM^ (Vector Laboratories, Newark, CA, USA), and nuclei were stained with 4,6-diamidino-2-phenylindole (Dapi; Merk Life Science). Confocal laser scanner microscopy was carried out by the Zeiss Confocal System and Zen software analysis (Zeiss, Oberkochen, Germany), and images were processed with Image Pro-Plus 6.0 (Media Cybernetics, Rockville, MA, USA). Controls for specificity included a lack of primary antibodies.

### Western blotting

Cells were lysed with RIPA buffer (0.05 mol/L Tris-HCl pH 7.7, 0.15 mol/L NaCl, 0.8% SDS, 10 mmol/L EDTA, 100 μM/L NaVO_4_, 50 mmol/L NaF, 0.3 mmol/L PMSF, and 5 mmol/L iodoacetic acid) containing protease inhibitors (all by Merk Life Science): leupeptin (50 μg/ml), aprotinin (5 μl/ml), and pepstatin (50 μg/ml). Cell lysates were then centrifuged at 12,500 rpm for 15 min at 4°C, and the supernatant protein content was determined using a BCA protein assay kit (Thermo Fisher). An equal amount of proteins (20–30 μg) was separated through SDS-PAGE electrophoresis and transferred to a nitrocellulose membrane (GE Healthcare, Milan, Italy). After blocking, the membranes were exposed to primary antibodies: mouse anti-cyclin D1 (1:1,000; #2926, Cell Signaling, Rehovot, Israel), mouse anti-CDK6 (1:1,000; #3136, Cell Signaling), rabbit anti-Snail (1:1,000; #3879, Cell Signaling), rabbit anti-vimentin (1:1,000; #5741, Cell Signaling), mouse anti-E-cadherin (1:1,000; sc-8426, Santa Cruz Biotechnology, Dallas, TX, USA), mouse anti-N-cadherin (1:1,000; sc-59987, Santa Cruz Biotechnology), mouse anti-alpha-tubulin (1:2,000; T6199, Merk Life Science), and rabbit anti-P0 (1:500; 10572-1-AP, Proteintech, Manchester, UK). Then, the membranes were incubated with HRP-conjugated anti-rabbit or anti-mouse secondary antibody for 1 h at room temperature and revealed using an enhanced chemiluminescence kit Cyanagen Ultra (Cyanagen, Bologna, Italy). In each experiment, tubulin expression was evaluated as a loading control. Blots were visualized using the Chemidoc MP Imaging System (Bio-Rad Laboratories) and analyzed by the Image Lab software (Bio-Rad Laboratories).

### *In vitro* cell proliferation and viability assays

Cells were plated in 35 mm Petri dishes and analyzed for viability and proliferation. All measurements were done by using the ImageJ 1.51 software (NIH, Bethesda, MD, USA). Approximately 6 × 10^4^ cells were plated and analyzed after 2, 3, 4, 5, 6, and 7 days *in vitro* (d.i.v.). To assess proliferation, the cells were collected with trypsin 0.05% and EDTA 0.02% in PBS (Merk Life Science), then suspended in DMEM (Euroclone, Pero, Italy), and counted with a hemocytometer. Viability was tested by the MTT [3-(4,5-dimethylthiazol-2-yl)-2,5-diphenyltetrazolium bromide; Merk Life Science] 0.5 mg/ml assay, for 30 min at 37°C. Absorbance was measured at 570 nm. Each experimental point was in quadruplicate, and experiments were replicated at least three times. Data were expressed as mean absorbance ± s.e.m.

### Wound healing and Boyden assay

A wound healing assay was performed to test migration. A scratch was made on the bottom of the Petri dish, then the medium was changed to a fresh medium, and the cells were exposed to DCP-LA 500 nM. Cells were then photographed with a scanning microscope (Axiovert 200, Zeiss) at different time points, i.e., 24, 48, and 72 h after the scratch. Pictures were acquired using the MetaVue software (Molecular Devices, Sunnyvale, CA, USA), and the areas between cell fronts were measured with Image-ProPlus 6.0 (MediaCybernetics), considering at least nine measurements. Data were calculated as the difference between the mean area at a specific time point and the area at time 0 (scratch time). Migration was also tested by the mean of Boyden chamber assay using a 48-well chamber, according to the manufacturer's instructions (Neuro Probe, Gaithersburg, MD, USA). In detail, cells were treated with DCP-LA for 1 and 6 h vs. control (pretreated). Then, the open-bottom wells of the chamber's upper compartment were filled with 10^5^ cells/well, collected by trypsin, and suspended in DMEM + 0.1 % BSA (Merk Life Science). In a series of samples, cells were treated with DCP-LA 500 mM also during the migration period (pretreated + treated cells). The bottom of the chamber was filled with 28 μl of DMEM+ FCS 1% as a chemoattractant. Cells migrate through a polyvinylpyrrolidone-free polycarbonate porous membrane (8 μm pores) pre-coated with gelatine (0.2 mg/ml in PBS, 5 days at 4°C). After migration (overnight, about 18 h at 37°C), cells adherent to the underside of the membrane were fixed by methanol and stained according to the Diff-Quik kit (Biomap, Milan, Italy). For quantitative analysis, three random objective fields were counted, each well, and the mean number of migrating cells was calculated.

### Statistical analysis

Data were statistically evaluated using the statistical package GraphPad Prism 6.00 (San Diego, USA), with independent or paired two-tailed samples *t*-test or one-way ANOVA followed by *post hoc* tests (see each figure legend for details). All data were expressed as mean ± s.e.m. of the determinations performed. Experiments were repeated at least three times. In pharmacological experiments, cell culture samples were randomly allocated to groups. Graphs were drawn with GraphPad Prism 6.00.

## Results

### SCs constitutively express PKCε

**Figure 1:**
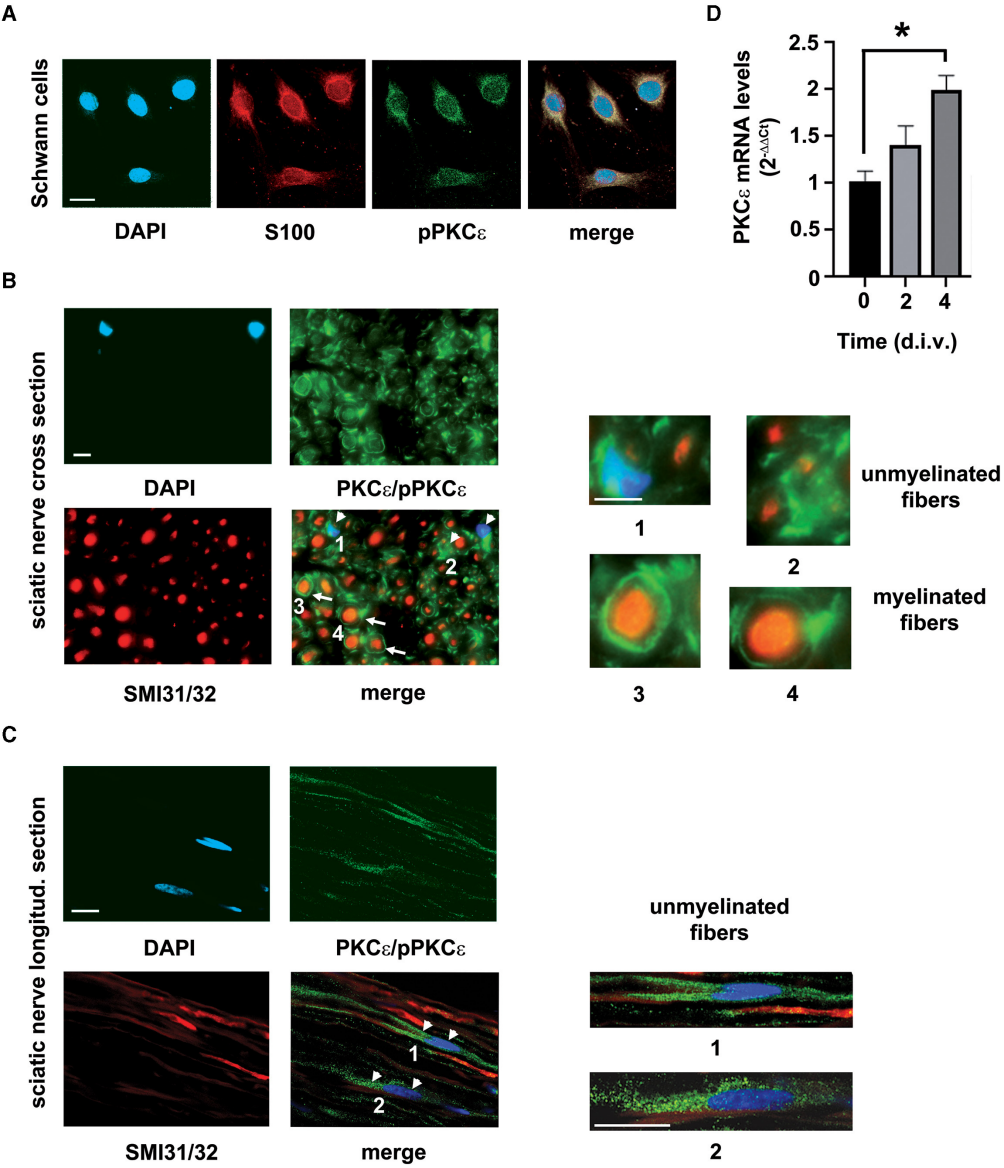
PKCε characterization in SCs. **(A)** IFL analysis demonstrated the presence of the active/phosphorylated form of PKCε (denoted in green) in rat SCs primary cultures. The characteristic marker S100 (in red) labeled SCs. DAPI (denoted in blue) stained nuclei. Merge images show co-localization (in yellowish) in SCs cytoplasm. Bar 10 μm. **(B)** Co-labeling of the active/phosphorylated form of PKCε (denoted in green) and neurofilament markers SMI31/32 (high-density neurofilament marker, denoted in red), coupled with morphology observation of myelinated and unmyelinated fibers, demonstrated that PKCε is constitutively expressed in peripheral nerve. Double labeling of cross sections of the sciatic nerve showed the presence of phosphorylated and non-phosphorylated PKCε in myelinating SCs (denoted as white arrows in merge; see also magnifications in panels 3, 4), as well as in unmyelinated fibers and in surrounding SCs (denoted as white arrowheads in merge; see magnifications in panels 1, 2). DAPI (denoted in blue) stained nuclei. Bar 10 μm. **(C)** Double labeling of longitudinal sections of the sciatic nerve showed the presence of phosphorylated and non-phosphorylated PKCε (denoted in green) in SCs (denoted as white arrowheads in merge) surrounding unmyelinated axons (neurofilament markers SMI31/32, denoted in red); see also magnifications in panels 1 and 2. DAPI (denoted in blue) stained nuclei. Bar 10 μm. **(D)** SCs culture conditions showed an upregulation of PKCε mRNA levels, significant at 4 days *in vitro* (d.i.v.) (*p < 0.01; ANOVA with Dunnett's test; *n* = 4; the experiment was repeated three times; data are mean ± s.e.m.).

To study the functional presence of PKCε in SCs, we assessed its expression in SCs *in vivo* and *in vitro*, respectively. In accordance with previous observations (Kawano et al., 1997; Bonalume et al., 2020), we confirmed that PKCε is constitutively expressed in SCs in culture (Figure 1A) and also in peripheral nerve, mostly localized in unmyelinated and in myelinated fibers (Figure 1B). In particular, double labeling of cross sections of the sciatic nerve with PKCε and SMI31/32 (high-density neurofilament marker) showed the presence of phosphorylated and non-phosphorylated PKCε in myelinating SCs (white arrows in Figure 1B merge and magnifications in panels 3, 4), as well as in unmyelinated axons and in surrounding SCs (white arrowheads in Figure 1B merge and magnifications in panels 1, 2). Staining for phosphorylated and non-phosphorylated PKCε was found also in SCs surrounding unmyelinated axons in the longitudinal nerve section (white arrowheads in Figure 1C and magnifications in panels 1 and 2). The PKCε gene expression was significantly upregulated (*p* < 0.05; Figure 1D) by the culture condition at 4 d.i.v.

### PKCε activation regulates SCs proliferation

**Figure 2:**
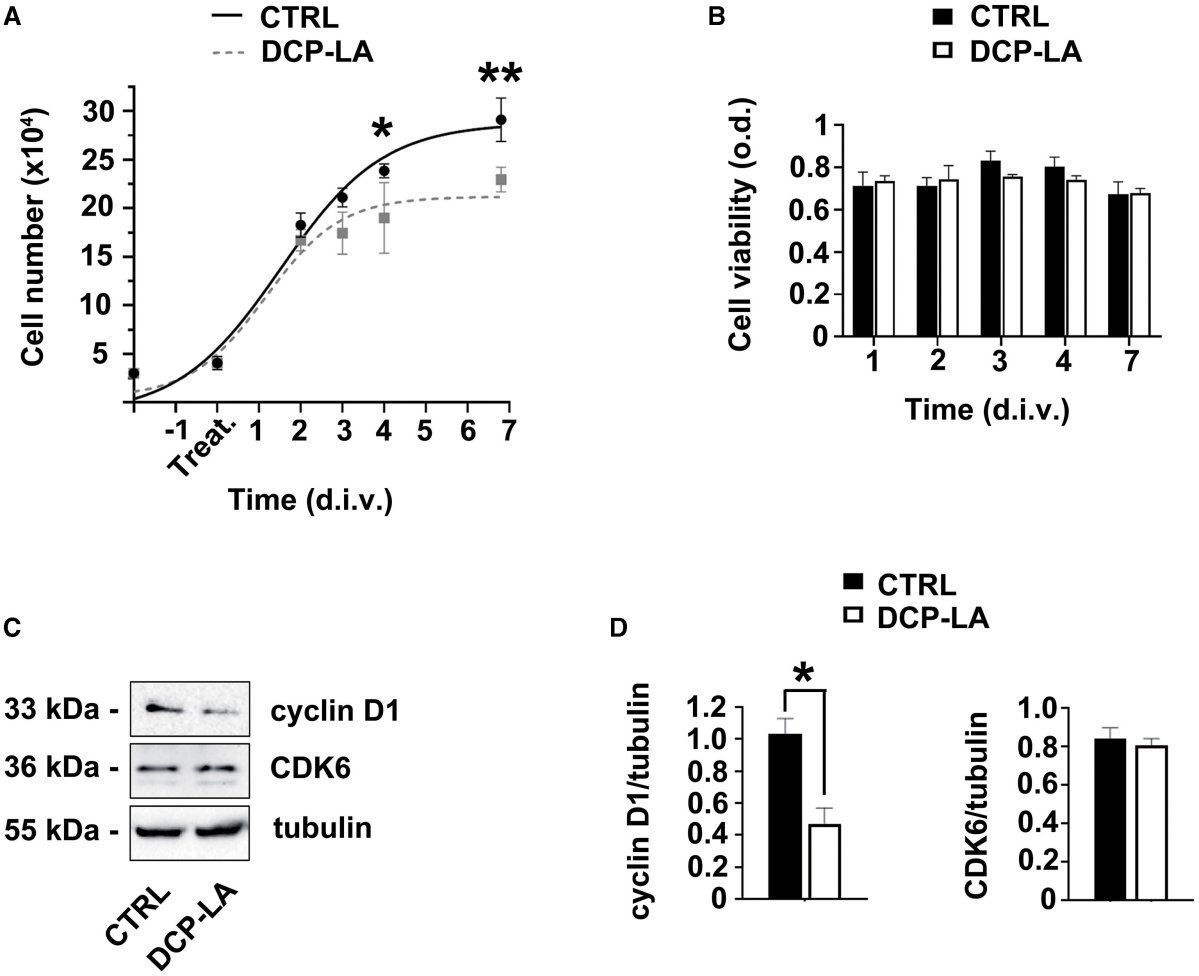
PKCε activation in SCs regulates proliferation. **(A)** SCs proliferation was assessed at 2, 3, 4, and 7 days *in vitro* (d.i.v.) following DCP-LA treatment 500 nM (dashed line). The activation of PKCε produced a significant decrease in cell proliferation at 4 (*p < 0.05) and 7 d.i.v. (**p < 0.01) vs. controls (CTRL). Experiments were repeated at least three times, and data were expressed as cell numbers (× 10^4^). Two-way ANOVA using Sidack's *post hoc* test was used for statistical analysis. **(B)** Percentage of SCs viability was assessed at 1, 2, 3, 4, and 7 d.i.v. following a DCP-LA 500 nM treatment (white columns) vs. control (CTRL, vehicle treated; black columns). No significant changes in SCs viability were observed. The values are means ± s.e.m. (*n* = 6). **(C)** Representative immunoblots of the cyclin D1/CDK6 complex following DCP-LA 500 nM treatment at 3 d.i.v. Tubulin was used as housekeeping; control SCs (CTRL) were treated with vehicle. **(D)** Quantitative analysis of cyclin D1 and CDK6 protein levels, normalized per tubulin, showed a significant decrease (*p < 0.05; Student's t-test; the experiment was repeated three times; data are mean ± s.e.m.) of cyclin D1 following 3 d.i.v.

The selective agonist DCP-LA was used to assess the effect of PKCε activation in SCs. Cell number was evaluated at 2, 3, 4, and 7 d.i.v. following treatment. We observed (Figure 2A) that the duplication rate was unchanged (identical slope) during the first 2 days of DCP-LA 500 nM treatment, while the proliferation was significantly decreased at 4 (*p* < 0.05) and 7 (*p* < 0.01) d.i.v. following DCP-LA 500 nM. Notably, this concentration is consistent with that used in previous experiments (Kawano et al., 1997; Bonalume et al., 2020). However, to exclude any possible toxic effect, we tested the DCP-LA on SCs viability. We found that the proliferation decrease was not associated with changes in cell viability; indeed, the MTT assay (Figure 2B) showed that DCP-LA 500 nM treatment does not modulate cell viability at any time points tested (1 to 7 d.i.v.). Taken together, these findings partially suggest a SC shift toward a differentiated state.

To support these results, the expression levels of some cell cycle regulatory proteins involved in G1/S transition were evaluated. Indeed, we analyzed the expression of the cyclin D1/CDK6 complex after treatment with DCP-LA 500 nM. Interestingly, immunoblots showed that cyclin D1 decreased at 3 d.i.v. after treatment, while CDK6 expression remained unchanged (Figure 2C). Quantitative blot analysis corroborated these data showing, above all, a significant drop (*p* < 0.05) of cyclin D1 at 3 d.i.v. (Figure 2D). Since cyclin D1/CDK6 complex has a role in regulating G1-phase progression and G1- to S-phase transition, this result supports the antiproliferative effect exerted by DCP-LA.

### PKCε activation increases SCs migration

**Figure 3:**
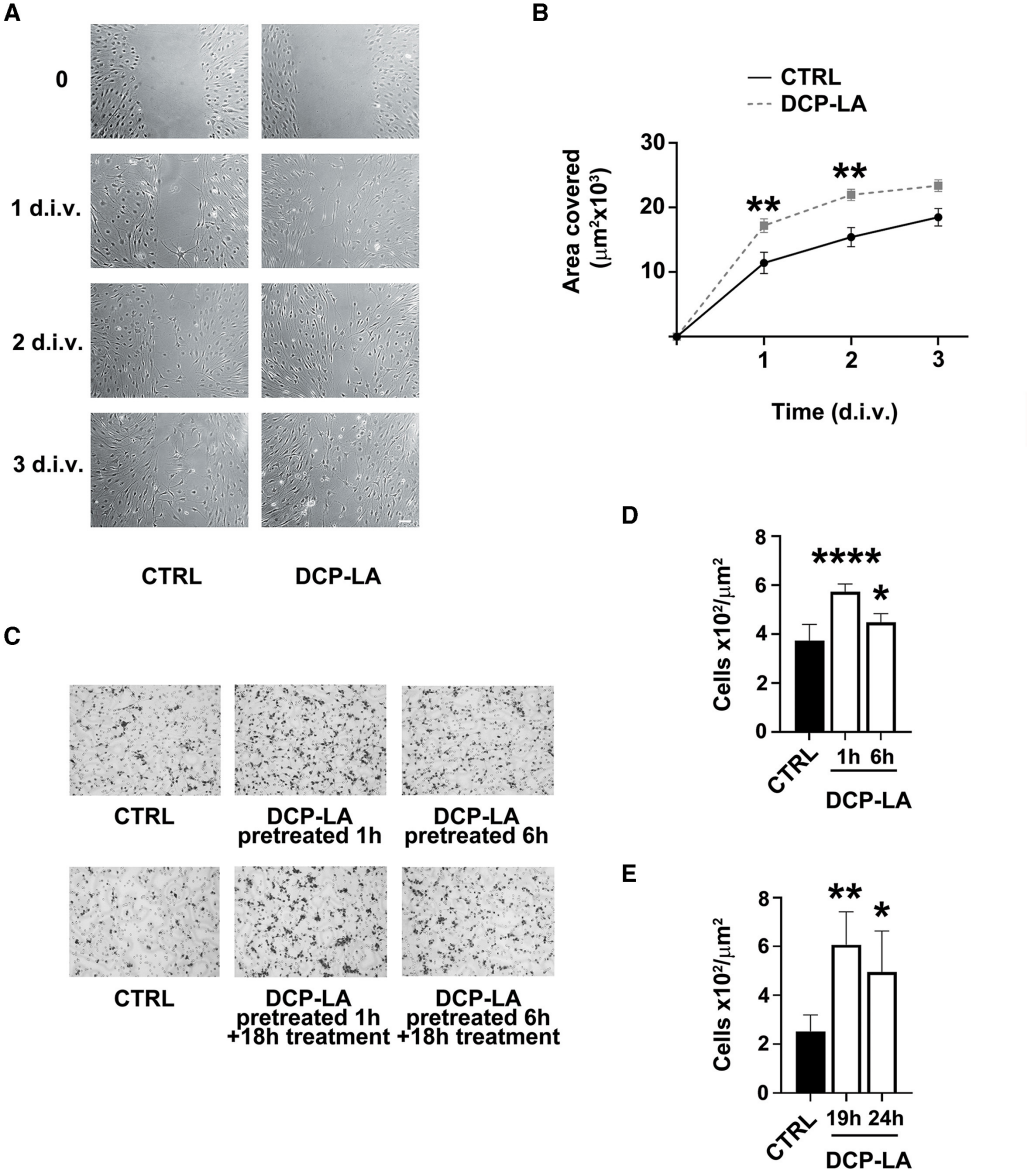
PKCε activation in SCs regulates migration. **(A)** Contrast-phase images of SCs cultures exposed to DCP-LA 500 nM for different times (1, 2, and 3 d.i.v.) compared to controls (CTRL), in which a scratch has been done on the bottom of the well (*t* = 0). SCs migrate repopulating the wounded region within 2 d.i.v., achieving a complete closure of the two sides at 3 d.i.v., while DCP-LA 500 nM speeded a complete closure as early as 1-2 d.i.v. Scale bar 30 μm. **(B)** Curves of the area covered by SCs (μm^2^ × 10^3^) exposed to DCP-LA 500 nM (dashed line) vs. controls (CTRL; vehicle). DCP-LA produced a significant increase in cell migration at 1 d.i.v. (**p < 0.01) and 2 d.i.v. (**p < 0.01). Two-way ANOVA using Sidack's *post-hoc* test was used for statistical analysis. The values are means ± s.e.m. (*n* = 4). **(C)** Boyden assay images of migrated SCs controls (CTRL) and pretreated for 1 and 6 h, respectively, to DCP-LA 500 nM. Cells underwent two different conditions: pretreatment for the reported time (pretreated) or continuously treated (pretreated+treated) that is exposed to DCP-LA also during the Boyden test migration (18 h). DCP-LA exposure made the SCs significantly responsive to the chemotactic agent FCS 1%, either when cells were pretreated only **(D)** or when pretreated cells were continuously exposed to DCP-LA also during the migration assay **(E)**, i.e., analyzed at 19 and 24 h, respectively. Migrating cells number (× 10^2^/μm^2^) increased significantly in all conditions: ****p < 0.0001, **p < 0.01, and *p < 0.05. The values are means ± s.e.m. (*n* = 4). One-way ANOVA using Tukey's *post hoc* test was used for all statistical analyses.

Since PKCε seemed to be involved in the partial EMT process, we assessed whether PKCε could affect also the SCs migration. We used a wound-healing assay on a cell monolayer to check for motility. In control condition (vehicle), SCs started repopulating the wounded region within 2 d.i.v., achieving a complete closure of the two sides at 3 d.i.v. (Figure 3A), while DCP-LA 500 nM treatment promoted almost completed closure as early as 1–2 d.i.v. Quantitative evaluation of the area covered by SCs (Figure 3B) indicates that DCP-LA induced a significant motility increase (*p* < 0.01) at 1 and 2 d.i.v. after treatment.

We next corroborated the migratory outcome of SCs by the mean of Boyden chamber assay (Figure 3C), following 1 or 6 h of pretreatment or after continuous treatment with DCP-LA 500 nM during migration. We found that DCP-LA significantly increased SCs migration following 1 h (*p* < 0.0001) and 6 h (*p* < 0.05) of pretreatment (Figure 3D). This effect was unchanged when SCs were continuously exposed to DCP-LA for the whole migration assay (19 h, *p* < 0.01 and 24 h, *p* < 0.05; Figure 3E), suggesting that the effect of DCP-LA on migration capability is retained independently by continuous exposure to the PKCε activator.

### Markers of SCs differentiation and EMT process are changed by PKCε activation

**Figure 4:**
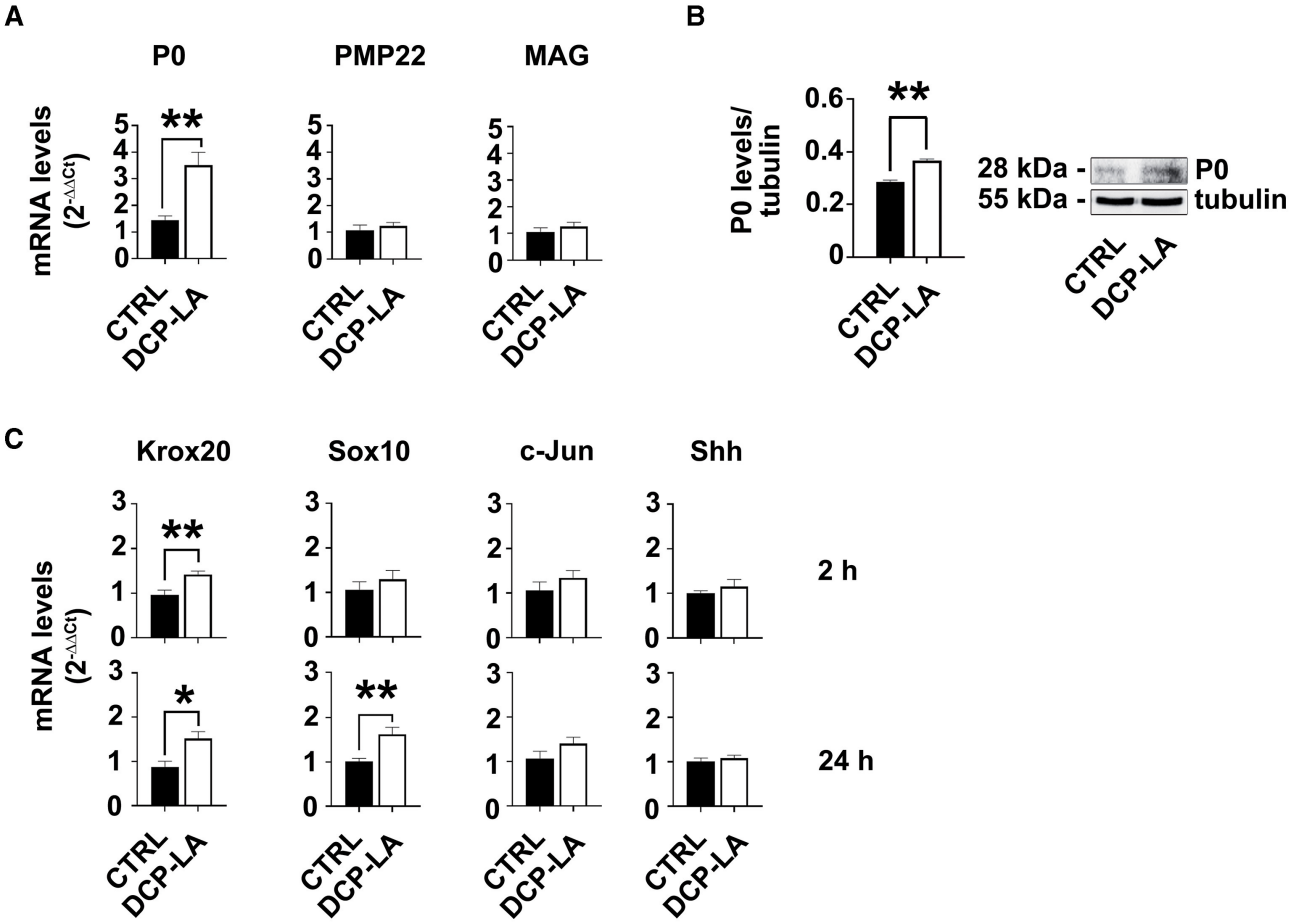
PKCε activation modulates SCs markers. **(A)** mRNA levels of the specific SCs marker P0 were significantly upregulated (**p < 0.01) at 24 h following DCP-LA 500 nM treatment, vs. control (CTRL, vehicle). PMP22 and MAG gene expression, indeed, did not change. The experiment was repeated three times, and values are means ± s.e.m. (*n* = 4). One-way ANOVA using Dunnett's *post-hoc* test was used for all statistical analyses. **(B)** Representative immunoblot and quantitative analysis of P0 protein levels, normalized per tubulin, confirmed a significant increase (**p < 0.01; Student's *t*-test; the experiment was repeated three times; data are mean ± s.e.m.) following 72 h DCP-LA 500 nM treatment, vs. control (CTRL, vehicle). **(C)** mRNA levels of the early genes Krox20 and Sox10, and markers of repairing SCs, c-Jun and Shh, respectively, were assessed at 2 h and 24 h following DCP-LA 500 nM treatment, vs. control (CTRL, vehicle). DCP-LA significantly increased Krox-20 gene expression at 2 h (**p < 0.01) and 24 h (*p < 0.05), as well as Sox10 at 24 h (**p < 0.01). The gene expression of c-Jun and Shh was unchanged at both the time points considered. The experiment was repeated three times and values are means ± s.e.m. (*n* = 4). One-way ANOVA using Dunnett's *post-hoc* test was used for all statistical analyses.

First, we tested the potential effect of PKCε activation on the expression of some characteristic myelin proteins, such as P0, peripheral myelin protein of 22kDa (PMP22), and myelin associated glycoprotein (MAG). At 24 h, P0 strongly responded to PKCε activation. Indeed, P0 gene expression was significantly increased (*p* < 0.01) following DCP-LA 500 nM treatment (Figure 4A). Similar result was obtained in western blot experiments, where DCP-LA significantly (*p* < 0.01) upregulated P0 protein levels at 72 h in primary SC cultures (Figure 4B). Conversely, PMP22 and MAG gene expression did not change (Figure 4A); myelin proteins expression did not change at other time points, i.e., 2 and 6 h (data not shown). Then, we analyzed whether PKCε also regulates the expression of some SC differentiation markers. In particular, we assessed the gene expression of two early genes of pro-myelinating/myelinating SCs (Krox20 and Sox10) (Jessen and Mirsky, 2019) and two markers of repairing SCs (c-Jun and Shh) (Jessen and Arthur-Farraj, 2019; Jessen and Mirsky, 2019) following DCP-LA 500 nM treatment. PKCε activation had a rapid and significative effect (*p* < 0.01) on Krox20 mRNA levels after 2 h of treatment, while the expression of other genes was unchanged (Figure 4C). Later, at 24 h post-treatment with DCP-LA 500 nM, the increased mRNA levels of Krox20 persisted (*p* < 0.05), whereas the gene expression of Sox10 was significantly increased (*p* < 0.01; Figure 4C). The gene expression of c-Jun and Shh, master regulators of the transition toward the repairing phenotype (Jessen et al., 2015), was unchanged at all the time points considered.

**Figure 5:**
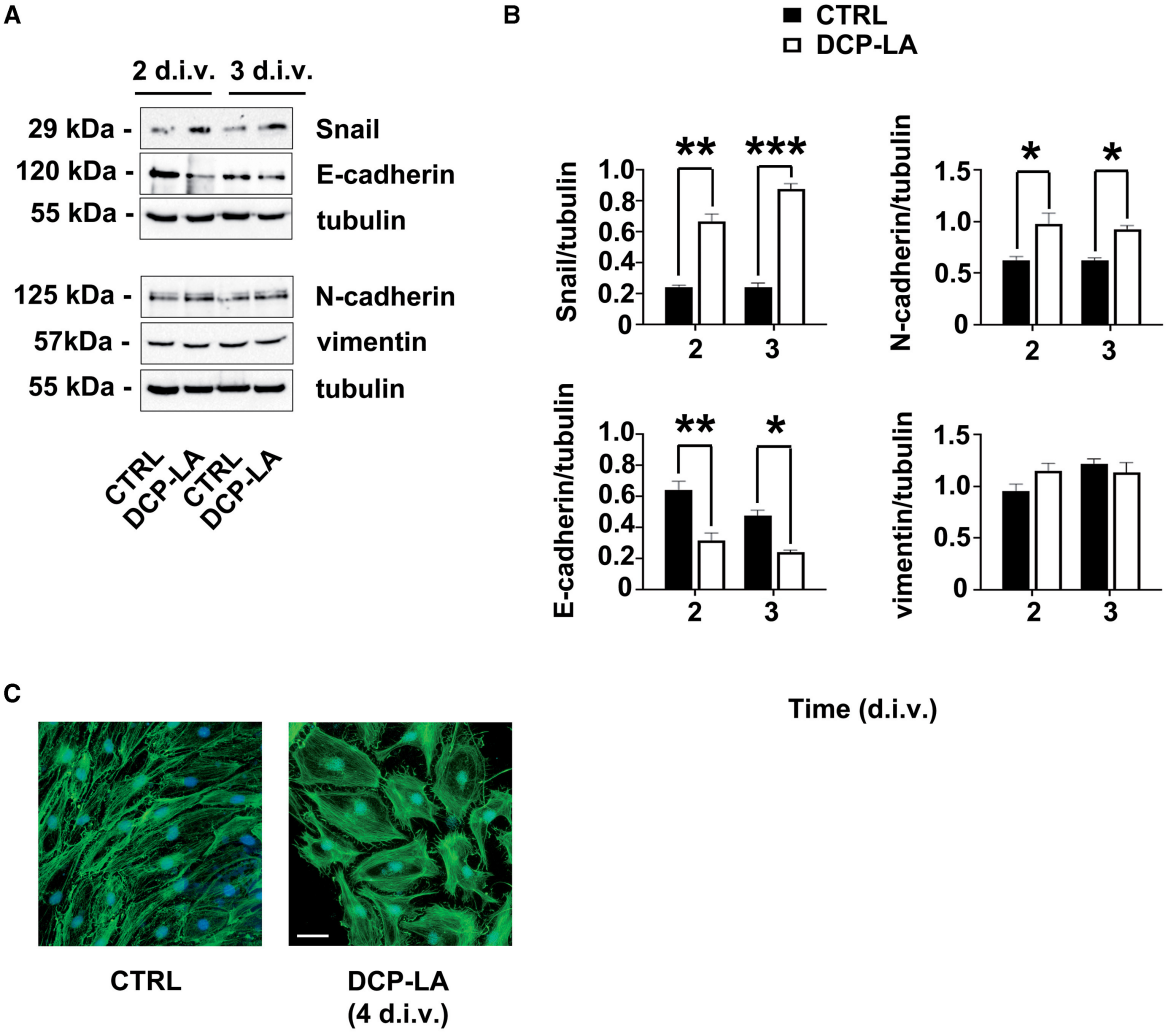
PKCε activation of the EMT-like process. **(A)** Representative immunoblots of specific markers of the EMT process (i.e. Snail; E-cadherin; N-cadherin; vimentin) following DCP-LA 500 nM treatment at 2 and 3 d.i.v. Tubulin was used as housekeeping; control SCs (CTRL) were treated with a vehicle. **(B)** Quantitative analysis of protein levels, normalized per tubulin, showing a significant increase of Snail at 2 d.i.v. (**p < 0.01) and 3 d.i.v. (***p < 0.001), and N-cadherin at 2 d.i.v. (*p < 0.05) and 3 d.i.v. (*p < 0.05) after DCP-LA, vs. control (CTRL, vehicle). Simultaneously, a significant decrease in E-cadherin levels was found at 2 (**p < 0.01) and 3 d.i.v. (*p < 0.05), respectively, after DCP-LA. Vimentin levels did not change at any time point considered. The experiment was repeated three times, and values are means ± s.e.m. (*n* = 4). One-way ANOVA using Bonferroni's *post hoc* test was used for all statistical analyses. **(C)** IFL for f-actin (phalloidin-FICT, in green) showing SCs morphologic rearrangements (at d.i.v. 4) in actin cytoskeleton toward an enlarged phenotype. Nuclei were stained with DAPI (in blue). Bar 20 μm.

Moreover, to characterize further the phenotype of SCs, we tested the expression of EMT markers after treatment with DCP-LA 500 nM. We found that the levels of the EMT-related transcription factor Snail were upregulated, and this rise repressed the E-cadherin protein expression (Figure 5A). Meanwhile, N-cadherin, but not vimentin levels, changed following DCP-LA 500 nM at the same time points (Figure 5A). Quantitative blot analysis corroborated these data showing, above all, a significant increase of Snail both at 2 (*p* < 0.01) and 3 d.i.v. (*p* < 0.001), respectively; at the same time, a significant decrease in E-cadherin levels was found at 2 (*p* < 0.01) and 3 d.i.v. (*p* < 0.05), respectively (Figure 5B). Finally, also N-cadherin levels increased significantly at 2 (*p* < 0.05) and 3 d.i.v. (*p* < 0.05), respectively (Figure 5B). In line with EMT-like changes, PKCε activation induced SCs' morphological rearrangement, turning to an enlarged phenotype, as evidenced by the f-actin label with phalloidin-FICT at 4 d.i.v. (Figure 5C).

### BDNF involvement in PKCε-mediated decrease of SCs proliferation

**Figure 6:**
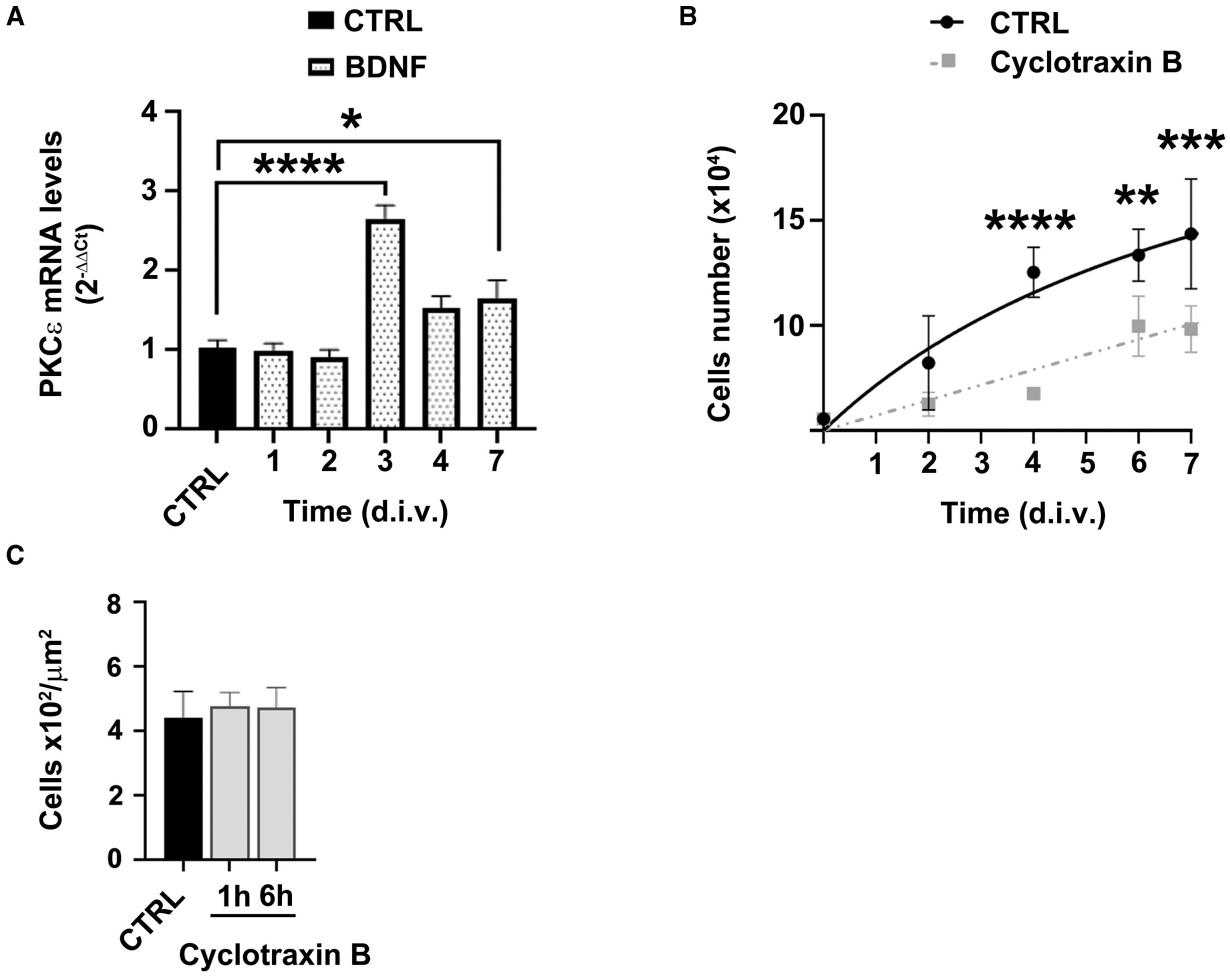
BDNF involvement in PKCε-mediated modulation of SCs proliferation and migration. **(A)** SCs exposure to BDNF (dot columns) induced a significant upregulation of PKCε mRNA levels, at 3 days *in vitro* (d.i.v.) (*p < 0.0001) and 7 d.i.v. (*p < 0.05; ANOVA with Dunnett's *post hoc* test; *n* = 4) vs. control (CTRL, vehicle). The experiment was repeated three times; data are mean ± s.e.m. **(B)** SCs proliferation was assessed at 2, 4, 6, and 7 days *in vitro* (d.i.v.) following cyclotraxin B 10 nM treatment (dashed line). The specific antagonism of TrkB produced a significant decrease in cell proliferation at 4 (****p < 0.0001), 6 (**p < 0.01), and 7 d.i.v. (***p < 0.001) vs. controls (CTRL). Experiments were repeated at least three times, and data were expressed as cell numbers (× 10^4^). Two-way ANOVA using Dunnett's *post-hoc* test was used for statistical analysis. **(C)** Cyclotraxin B 10 nM exposure did not change SCs migration, vs. controls (CTRL, vehicle), neither at 1 h nor at 6 h of treatment. The values of migrating cells number (× 10^2^/μm^2^) are means ± s.e.m. (*n* = 4). One-way ANOVA using Tukey's *post hoc* test was used for all statistical analyses.

Our previous observations indicated that SCs tonically release ALLO and, in turn, BDNF, which activates PKCε in DRG sensory neurons (Bonalume et al., 2020). Indeed, BDNF is secreted from SCs in a range of picomolar concentrations (Bonalume et al., 2020). On this basis, we tested the hypothesis of whether BDNF might also regulate PKCε in SCs. As shown in Figure 6A, exogenous BDNF 1 nM significantly upregulated the PKCε expression after 3 (*p* < 0.0001) and 7 (*p* < 0.05) days in culture, respectively. Therefore, the logical next step was to investigate whether the effects of PKCε activation on SCs proliferation, described above, might be the consequence of BDNF modulation. To test this hypothesis, we used the selective BDNF/TrkB antagonist cyclotraxin B 10 nM. As expected, cyclotraxin B significantly decreased SCs proliferation after 4 (*p* < 0.0001), 6 (*p* < 0.01), and 7 d.i.v. (*p* < 0.001), respectively (Figure 6B). No changes in SC migration were observed, either when the SCs were exposed to cyclotraxin B for 1 and 6 h of pretreatment (Figure 6C). These results suggested that the effects of PKCε activation on SCs proliferation might be ascribed to an autocrine modulation *via* BDNF.

## Discussion

**Figure 7:**
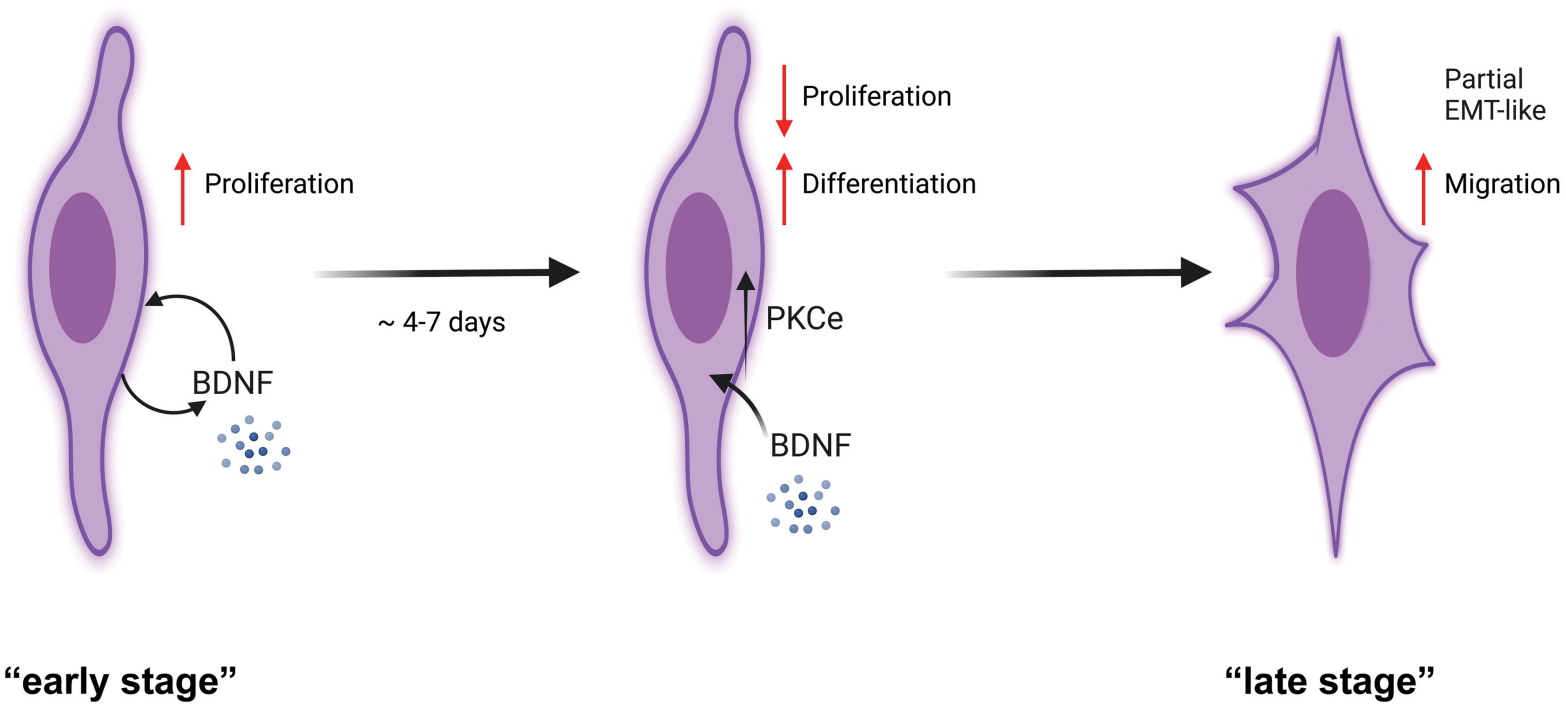
Schematic representation of PKCε activation effects in SCs. SCs at a potential “early stage” of culture express an autocrine mechanism of proliferation through BDNF synthesis and release (Luo et al., 2014; Bonalume et al., 2020). We showed that SCs express PKCε under the BDNF control. PKCε activation decreased SCs proliferation, switching the cells toward a more differentiated state. As a consequence, at the “late stage” of culture, the PKCε activation promoted a partial EMT-like process, increasing migration. We propose that PKCε activation is the switching-off mechanism by which BDNF controls SCs proliferation.

Our experiments demonstrated that PKCε is expressed and active in SCs of the PNS. In detail, we found that (Figure 7) SCs express PKCε under the control of autocrine BDNF and TrkB, and PKCε activation decreased SCs proliferation, switching the cells toward a more differentiated state. We propose that PKCε activation is a kind of switching-off mechanism by which BDNF regulates SCs proliferation. Concurrently, after some days of SCs *in vitro*, PKCε activation also promoted a partial EMT-like process, increasing migration and controlling SCs plasticity.

A compelling line of evidence indicated that, in the PNS, BDNF is synthesized and released by neurons (Apfel et al., 1996; Cho et al., 1997) and SCs (Luo et al., 2014; Bonalume et al., 2020). In the PNS, BDNF plays an important role in promoting axonal regeneration and remyelination when SCs were transplanted into nerve injuries (Bamber et al., 2001; Tep et al., 2012). Axonal BDNF binds to SCs p75 neurotrophin receptor promoting early stages of myelination (Cosgaya et al., 2002; Chan et al., 2006). Then, at later stages following myelination, BDNF binds to TrkB on SCs and limits myelination (Xiao et al., 2009), fulfilling a neuron-to-SCs crosstalk mechanism. However, BDNF seems to exert a mutual bidirectional control on the glial and neuronal compartment of the PNS. Indeed, BDNF has a role in the SCs-to-neuron crosstalk, through a TrkB receptor, so that BDNF activates PKCε in DRG sensory neurons, likely participating in the control of pain onset and chronification (Bonalume et al., 2020).

Here, we showed that BDNF possesses also an autocrine function in SCs, regulating proliferation and inducing, at the same time, PKCε, for further later inhibitory control of proliferation. Indeed, BDNF significantly upregulated PKCε expression 3 days after treatment, in accordance with the decrease in SCs proliferation observed 3 days after PKCε activation. It was not surprising to see that at shorter times, i.e., 24 h, BDNF did not change PKCε levels, in line with the unchanged SCs proliferation at a shorter time following PKCε activation. Moreover, proof of the autocrine activity of BDNF *via* PKCε is also supported by the observed increase in PKCε levels under basal culture conditions (see Figure 1D), which becomes statistically significant after 4 d.i.v. (as expected, given the low endogenous BDNF concentration), and the concomitant slowing in SCs proliferation, following the block of the TrkB receptor with cyclotraxin B.

This antiproliferative feature is corroborated by the analysis of cyclin-D1-CDK6 complex. Indeed, the decrease in cyclin-D1 and the subsequent lack of CDK6 activation support the block of SCs in the G1 phase of the cell cycle. The G1-S transition is important for mitogenic activity. CDK6 belongs to a family of protein kinases that coordinate the progression through several phases of the cell cycle (in particular for entry into the S phase), *via* their regulatory subunits, the cyclins like cyclin-D1 (Kohn, 1999; Roberts, 1999).

The effect of PKCε activation on the early genes, first on Krox20 and then on Sox10, evidenced a promotion of a more differentiated state of SCs. Established *in vivo* evidence stated that Krox20 plays a crucial role in PNS myelination (Le et al., 2005) and synergizes with Sox10 (Svaren and Meijer, 2008). Therefore, our findings are in accordance with the literature, evidencing an induction of Krox20 on the P0 expression *in vitro* (Parkinson et al., 2004). Although SCs are an example of plastic and adaptive cells, spanning among some differentiated stages, PKCε seems to be not involved in the repair SCs (i.e., SCs activated during injury response), since the characteristic transcription factors c-Jun and Shh were unchanged. Avowed proofs indicated that Krox20 antagonizes c-Jun expression (Parkinson et al., 2004, 2008).

Our results evidenced that SCs also undergo a partial EMT-like process. EMT is a process by which cells gradually lose their epithelial characteristics, like cell–cell adhesion and polarity, and gain mesenchymal ones, such as spindle-shaped morphology and migration ability (Thiery et al., 2009; Savagner, 2010). However, EMT is a dynamic process that does not necessarily drive cells toward a fully phenotypical state but rather toward hybrid states named partial EMT (Verstappe and Berx, 2023). Partial EMT typically provides cells with increased motility and morphological flexibility.

Partial EMT has been identified in injured nerves of the PNS (Arthur-Farraj et al., 2017; Clements et al., 2017) highlighting how the plasticity of SCs follows a complex mechanism. Compelling evidence indicated that PKCε promotes EMT in different cancers, i.e., breast, prostate or head, and neck squamous cell carcinoma (Gandellini et al., 2009; Jain and Basu, 2014), whereas PKCε expression correlates with tumor grade and poor disease outcome (Pan et al., 2005, 2006; Aziz et al., 2007). Interestingly, the growth factors (e.g., TGF-beta, PDGF, and EGF) are prominent inducers of EMT (Holz et al., 2011; Katsuno et al., 2013; Wu et al., 2013), whereas the BDNF/TrkB pathway proved effective modulator of EMT in different cells and cancer progression (Dudas et al., 2011; Serafim Junior et al., 2020; Moriwaki et al., 2022; Tian et al., 2023).

The drop of the adhesion protein through the reduction of E-cadherin is a prominent feature of EMT and a sign of loss of cell-to-cell contact (Thiery et al., 2009), which is, in accordance with the observed increase in migration, found in our SCs model. Snail upregulation, indeed, was expected, since it negatively regulates E-cadherin (Peinado et al., 2004). Snail (Snai1) is a zinc finger transcription factor that usually acts as a gene repressor and plays a key role in EMT regulation. Snail exerts its effects by decreasing the E-cadherin expression by binding to its promoter (Peinado et al., 2004). N-cadherin and vimentin, instead, are markers of immature SCs (Soto and Monje, 2017). Although N-cadherin expression was changed following PKCε activation, vimentin levels were unmodified by the treatment, supporting the existence of a partial EMT-like process in SCs.

Perineural invasion is a process of cancer cell invasion in, around, and through the nerves, observed in salivary adenoid cystic carcinoma (SACC). A recent paper by Shan et al. (2016) showed that SCs might induce SACC cells to differentiate into SC-like cells *via* the BDNF/TrkB axis. Overall, this supports the general role of SCs to promote EMT through the BDNF/TrkB axis.

We propose that PKCε activation is the switching-off mechanism by which BDNF controls SCs proliferation. From another side, it could be that BDNF is essential in promoting SCs proliferation at the beginning of SCs *in vitro*, acting as an autocrine factor, then SCs use the PKCε pathways to counteract BDNF proliferating control, inducing differentiation. However, partial EMT and migration seem to follow other regulatory mechanisms that are still to be elucidated.

Studies of the signals mediating the balance between proliferation, differentiation, and migration have revealed a number of factors important for cellular decision-making, which at different cellular stages modulate the overlapping presence of such cellular processes. Previously, we highlighted that SCs synthesize and release the neuroactive steroid ALLO (Faroni and Magnaghi, 2011; Bonalume et al., 2020; Colciago et al., 2020), which, through an autocrine mechanism, upregulates the BDNF synthesis and release. In turn, the SCs-released BDNF activates PKCε in sensory neurons, likely participating in the control of pain (Bonalume et al., 2020). We do not exclude that the SCs' release of ALLO and the subsequent autocrine control of BDNF have additional effects on the SCs fate *per se*. In accordance, and based on the aforementioned results, here we suggest one alternative mechanism through which PKCε activation in SCs diminishes BDNF-induced proliferation and switch SCs toward a partial EMT transition and a migratory feature. In other words, PKCε is a sort of escape mechanism, which controls proliferation at longer *in vitro* cellular conditions, and induces SCs differentiation toward a partial EMT phenotype. Here we posit the hypothesis of a novel regulatory system for SCs fate and plasticity, which might take importance in those cellular processes controlling plasticity and potentially tumorigenesis. Much emerging evidence, indeed, has confirmed the crosstalk between the cancer microenvironment and PNS (Zahalka et al., 2017; Monje et al., 2020), pointing out the importance of SCs in supporting cancer progression.

## Data availability statement

The raw data supporting the conclusions of this article will be made available by the authors, without undue reservation.

## Ethics statement

The animal study was reviewed and approved by the Ethics Committee University of Milan.

## Author contributions

TM, AC, RM, and MM performed all experiments, acquired the microscope images, and analyzed all of the data. VM conceived and designed the experiments, provided guidance, wrote the manuscript, and analyzed the data. All authors contributed to writing and read and approved the final version of the manuscript.

## References

[B1] Aksoy E. Goldman M. Willems F. (2004). Protein kinase C epsilon: a new target to control inflammation and immune-mediated disorders. Int. J. Biochem. Cell. Biol. 36, 183–188. 10.1016/S1357-2725(03)00210-3 14643884

[B2] Aley K. O. Messing R. O. Mochly-Rosen D. Levine J. D. (2000). Chronic hypersensitivity for inflammatory nociceptor sensitization mediated by the epsilon isozyme of protein kinase C. J. Neurosci. 20, 4680–4685. 10.1523/JNEUROSCI.20-12-04680.2000 10844037 PMC6772473

[B3] Amadesi S. Cottrell G. S. Divino L. Chapman K. Grady E. F. Bautista F.. (2006). Protease-activated receptor 2 sensitizes TRPV1 by protein kinase Cepsilon- and A-dependent mechanisms in rats and mice. J. Physiol. 575, 555–571. 10.1113/jphysiol.2006.111534 16793902 PMC1819458

[B4] Apfel S. C. Wright D. E. Wiideman A. M. Dormia C. Snider W. D. Kessler J. A.. (1996). Nerve growth factor regulates the expression of brain-derived neurotrophic factor mRNA in the peripheral nervous system. Mol. Cell Neurosci. 7, 134–142. 10.1006/mcne.1996.0010 8731481

[B5] Arthur-Farraj P. J. Morgan C. C. Adamowicz M. Gomez-Sanchez J. A. Fazal S. V. Beucher A.. (2017). Changes in the coding and non-coding transcriptome and DNA methylome that define the schwann cell repair phenotype after nerve injury. Cell. Rep. 20, 2719–2734. 10.1016/j.celrep.2017.08.064 28903050 PMC5608958

[B6] Aziz M. H. Manoharan H. T. Church D. R. Dreckschmidt N. E. Zhong W. Oberley T. D.. (2007). Protein kinase Cepsilon interacts with signal transducers and activators of transcription 3 (Stat3), phosphorylates Stat3Ser727, and regulates its constitutive activation in prostate cancer. Cancer Res 67, 8828–8838. 10.1158/0008-5472.CAN-07-1604 17875724

[B7] Bamber N. I. Li H. Lu X. Oudega M. Aebischer P. Xu X. M.. (2001). Neurotrophins BDNF and NT-3 promote axonal re-entry into the distal host spinal cord through Schwann cell-seeded mini-channels. Eur. J. Neurosci. 13, 257–268. 10.1046/j.1460-9568.2001.01387.x 11168530

[B8] Bogen O. Alessandri-Haber N. Chu C. Gear R. W. Levine J. D. (2012). Generation of a pain memory in the primary afferent nociceptor triggered by PKCepsilon activation of CPEB. J. Neurosci. 32, 2018–2026. 10.1523/JNEUROSCI.5138-11.2012 22323716 PMC3305286

[B9] Bonalume V. Caffino L. Castelnovo L. F. Faroni A. Giavarini F. Liu S.. (2020). Schwann cell autocrine and paracrine regulatory mechanisms, mediated by allopregnanolone and BDNF, modulate PKC epsilon in peripheral sensory neurons. Cells 9, 874. 10.3390/cells9081874 32796542 PMC7465687

[B10] Chan J. R. Jolicoeur C. Yamauchi J. Elliott J. Fawcett J. P. Ng B. K.. (2006). The polarity protein Par-3 directly interacts with p75NTR to regulate myelination. Science 314, 832–836. 10.1126/science.1134069 17082460

[B11] Cho H. J. Kim S. Y. Park M. J. Kim D. S. Kim J. K. Chu M. Y.. (1997). Expression of mRNA for brain-derived neurotrophic factor in the dorsal root ganglion following peripheral inflammation. Brain Res. 749, 358–362. 10.1016/S0006-8993(97)00048-6 9138740

[B12] Clements M. P. Byrne E. Camarillo Guerrero L. F. Cattin A. L. Zakka L. Ashraf A.. (2017). The wound microenvironment reprograms schwann cells to invasive mesenchymal-like cells to drive peripheral nerve regeneration. Neuron 96, 98–114. 10.1016/j.neuron.2017.09.008 28957681 PMC5626803

[B13] Colciago A. Bonalume V. Melfi V. Magnaghi V. (2020). Genomic and non-genomic action of neurosteroids in the peripheral nervous system. Front. Neurosci. 14, 796. 10.3389/fnins.2020.00796 32848567 PMC7403499

[B14] Cosgaya J. M. Chan J. R. Shooter E. M. (2002). The neurotrophin receptor p75NTR as a positive modulator of myelination. Science 298, 1245–1248. 10.1126/science.1076595 12424382

[B15] Dongre A. Weinberg R. A. (2019). New insights into the mechanisms of epithelial-mesenchymal transition and implications for cancer. Nat. Rev. Mol. Cell Biol. 20, 69–84. 10.1038/s41580-018-0080-4 30459476

[B16] Dudas J. Bitsche M. Schartinger V. Falkeis C. Sprinzl G. M. Riechelmann H.. (2011). Fibroblasts produce brain-derived neurotrophic factor and induce mesenchymal transition of oral tumor cells. Oral. Oncol. 47, 98–103. 10.1016/j.oraloncology.2010.11.002 21147546 PMC3042593

[B17] Faroni A. Magnaghi V. (2011). The neurosteroid allopregnanolone modulates specific functions in central and peripheral glial cells. Front. Endocrinol. 2, 103. 10.3389/fendo.2011.00103 22654838 PMC3356145

[B18] Ferrari L. F. Araldi D. Levine J. D. (2015). Distinct terminal and cell body mechanisms in the nociceptor mediate hyperalgesic priming. J. Neurosci. 35, 6107–6116. 10.1523/JNEUROSCI.5085-14.2015 25878283 PMC4397607

[B19] Gandellini P. Folini M. Longoni N. Pennati M. Binda M. Colecchia M.. (2009). miR-205 Exerts tumor-suppressive functions in human prostate through down-regulation of protein kinase Cepsilon. Cancer Res. 69, 2287–2295. 10.1158/0008-5472.CAN-08-2894 19244118

[B20] Gonzalez S. L. Meyer L. Raggio M. C. Taleb O. Coronel M. F. Patte-Mensah C.. (2019). Allopregnanolone and progesterone in experimental neuropathic pain: former and new insights with a translational perspective. Cell. Mol. Neurobiol. 39, 523–537. 10.1007/s10571-018-0618-1 30187261 PMC11469882

[B21] Gorin M. A. Pan Q. (2009). Protein kinase C epsilon: an oncogene and emerging tumor biomarker. Mol. Cancer 8, 9. 10.1186/1476-4598-8-9 19228372 PMC2647895

[B22] Hodge C. W. Mehmert K. K. Kelley S. P. Mcmahon T. Haywood A. Olive M. F.. (1999). Supersensitivity to allosteric GABA(A) receptor modulators and alcohol in mice lacking PKCepsilon. Nat. Neurosci. 2, 997–1002. 10.1038/14795 10526339

[B23] Holz C. Niehr F. Boyko M. Hristozova T. Distel L. Budach V.. (2011). Epithelial-mesenchymal-transition induced by EGFR activation interferes with cell migration and response to irradiation and cetuximab in head and neck cancer cells. Radiother Oncol. 101, 158–164. 10.1016/j.radonc.2011.05.042 21665310

[B24] Iser I. C. Lenz G. Wink M. R. (2019). EMT-like process in glioblastomas and reactive astrocytes. Neurochem Int. 122, 139–143. 10.1016/j.neuint.2018.11.016 30496766

[B25] Jain K. Basu A. (2014). Protein Kinase C-epsilon promotes EMT in breast cancer. Breast Cancer. 8, 61–67. 10.4137/BCBCR.S13640 24701121 PMC3972078

[B26] Jessen K. R. Arthur-Farraj P. (2019). Repair Schwann cell update: adaptive reprogramming, EMT, and stemness in regenerating nerves. Glia 67, 421–437. 10.1002/glia.23532 30632639

[B27] Jessen K. R. Mirsky R. (2019). The success and failure of the schwann cell response to nerve injury. Front. Cell Neurosci. 13, 33. 10.3389/fncel.2019.00033 30804758 PMC6378273

[B28] Jessen K. R. Mirsky R. Arthur-Farraj P. (2015). The role of cell plasticity in tissue repair: adaptive cellular reprogramming. Dev. Cell 34, 613–620. 10.1016/j.devcel.2015.09.005 26418293

[B29] Katsuno Y. Lamouille S. Derynck R. (2013). TGF-beta signaling and epithelial-mesenchymal transition in cancer progression. Curr. Opin. Oncol. 25, 76–84. 10.1097/CCO.0b013e32835b6371 23197193

[B30] Kawano S. Okajima S. Mizoguchi A. Tamai K. Hirasawa Y. Ide C.. (1997). Immunocytochemical distribution of Ca(2+)-independent protein kinase C subtypes (delta, epsilon, and zeta) in regenerating axonal growth cones of rat peripheral nerve. Neuroscience 81, 263–273. 10.1016/S0306-4522(97)00158-9 9300419

[B31] Khasar S. G. Lin Y. H. Martin A. Dadgar J. Mcmahon T. Wang D.. (1999). A novel nociceptor signaling pathway revealed in protein kinase C epsilon mutant mice. Neuron 24, 253–260. 10.1016/S0896-6273(00)80837-5 10677042 PMC11587340

[B32] Kohn K. W. (1999). Molecular interaction map of the mammalian cell cycle control and DNA repair systems. Mol. Biol. Cell 10, 2703–2734. 10.1091/mbc.10.8.2703 10436023 PMC25504

[B33] Le N. Nagarajan R. Wang J. Y. Araki T. Schmidt R. E. Milbrandt J.. (2005). Analysis of congenital hypomyelinating Egr2Lo/Lo nerves identifies Sox2 as an inhibitor of Schwann cell differentiation and myelination. Proc. Natl. Acad. Sci. U S A 102, 2596–2601. 10.1073/pnas.0407836102 15695336 PMC548989

[B34] Luo B. Huang J. Lu L. Hu X. Luo Z. Li M.. (2014). Electrically induced brain-derived neurotrophic factor release from Schwann cells. J. Neurosci. Res. 92, 893–903. 10.1002/jnr.23365 24753179

[B35] Magnaghi V. Veiga S. Ballabio M. Gonzalez L. C. Garcia-Segura L. M. Melcangi R. C.. (2006). Sex-dimorphic effects of progesterone and its reduced metabolites on gene expression of myelin proteins by rat Schwann cells. J. Peripher. Nerv. Syst. 11, 111–118. 10.1111/j.1085-9489.2006.00075.x 16787508

[B36] Mauro N. Manfredi A. Ranucci E. Procacci P. Laus M. Antonioli D.. (2013). Degradable poly(amidoamine) hydrogels as scaffolds for in vitro culturing of peripheral nervous system cells. Macromol. Biosci. 13, 332–347. 10.1002/mabi.201200354 23239646

[B37] Melfi S. Montt Guevara M. M. Bonalume V. Ruscica M. Colciago A. Simoncini T.. (2017). Src and phospho-FAK kinases are activated by allopregnanolone promoting Schwann cell motility, morphology and myelination. J. Neurochem. 141, 165–178. 10.1111/jnc.13951 28072455

[B38] Monje M. Borniger J. C. D'silva N. J. Deneen B. Dirks P. B. Fattahi F. (2020). Roadmap for the emerging field of cancer neuroscience. Cell 181, 219–222. 10.1016/j.cell.2020.03.034 32302564 PMC7286095

[B39] Moriwaki K. Wada M. Kuwabara H. Ayani Y. Terada T. Higashino M.. (2022). BDNF/TRKB axis provokes EMT progression to induce cell aggressiveness via crosstalk with cancer-associated fibroblasts in human parotid gland cancer. Sci. Rep. 12, 17553. 10.1038/s41598-022-22377-9 36266462 PMC9584965

[B40] Pan Q. Bao L. W. Kleer C. G. Sabel M. S. Griffith K. A. Teknos T. N.. (2005). Protein kinase C epsilon is a predictive biomarker of aggressive breast cancer and a validated target for RNA interference anticancer therapy. Cancer Res. 65, 8366–8371. 10.1158/0008-5472.CAN-05-0553 16166314

[B41] Pan Q. Bao L. W. Teknos T. N. Merajver S. D. (2006). Targeted disruption of protein kinase C epsilon reduces cell invasion and motility through inactivation of RhoA and RhoC GTPases in head and neck squamous cell carcinoma. Cancer Res. 66, 9379–9384. 10.1158/0008-5472.CAN-06-2646 17018591 PMC4383316

[B42] Parkinson D. B. Bhaskaran A. Arthur-Farraj P. Noon L. A. Woodhoo A. Lloyd A. C.. (2008). c-Jun is a negative regulator of myelination. J. Cell Biol. 181, 625–637. 10.1083/jcb.200803013 18490512 PMC2386103

[B43] Parkinson D. B. Bhaskaran A. Droggiti A. Dickinson S. D'antonio M. Mirsky R. (2004). Krox-20 inhibits Jun-NH2-terminal kinase/c-Jun to control Schwann cell proliferation and death. J Cell. Biol. 164, 385–394. 10.1083/jcb.200307132 14757751 PMC2172235

[B44] Peinado H. Ballestar E. Esteller M. Cano A. (2004). Snail mediates E-cadherin repression by the recruitment of the Sin3A/histone deacetylase 1 (HDAC1)/HDAC2 complex. Mol. Cell. Biol. 24, 306–319. 10.1128/MCB.24.1.306-319.2004 14673164 PMC303344

[B45] Puia G. Ravazzini F. Castelnovo L. F. Magnaghi V. (2015). PKCepsilon and allopregnanolone: functional cross-talk at the GABAA receptor level. Front. Cell. Neurosci. 9, 83. 10.3389/fncel.2015.00083 25852476 PMC4365694

[B46] Qi Z. H. Song M. Wallace M. J. Wang D. Newton P. M. Mcmahon T.. (2007). Protein kinase C epsilon regulates gamma-aminobutyrate type A receptor sensitivity to ethanol and benzodiazepines through phosphorylation of gamma2 subunits. J. Biol. Chem. 282, 33052–33063. 10.1074/jbc.M707233200 17875639

[B47] Roberts J. M. (1999). Evolving ideas about cyclins. Cell 98, 129–132. 10.1016/S0092-8674(00)81007-7 10428024

[B48] Savagner P. (2010). The epithelial-mesenchymal transition (EMT) phenomenon. Ann. Oncol. 21, vii89–vii92. 10.1093/annonc/mdq292 PMC3379967 20943648

[B49] Serafim Junior V. Fernandes G. M. M. Oliveira-Cucolo J. G. Pavarino E. C. Goloni-Bertollo E. M. (2020). Role of Tropomyosin-related kinase B receptor and brain-derived neurotrophic factor in cancer. Cytokine 136, 155270. 10.1016/j.cyto.2020.155270 32911446

[B50] Shan C. Wei J. Hou R. Wu B. Yang Z. Wang L.. (2016). Schwann cells promote EMT and the Schwann-like differentiation of salivary adenoid cystic carcinoma cells via the BDNF/TrkB axis. Oncol. Rep. 35, 427–435. 10.3892/or.2015.4366 26530352

[B51] Shirai Y. Adachi N. Saito N. (2008). Protein kinase Cepsilon: function in neurons. FEBS J 275, 3988–3994. 10.1111/j.1742-4658.2008.06556.x 18637121

[B52] Soto J. Monje P. V. (2017). Axon contact-driven Schwann cell dedifferentiation. Glia 65, 864–882. 10.1002/glia.23131 28233923 PMC5395415

[B53] Srinivasan R. Wolfe D. Goss J. Watkins S. Groat D. e Sculptoreanu W. C. A. Glorioso J.C. (2008). Protein kinase C epsilon contributes to basal and sensitizing responses of TRPV1 to capsaicin in rat dorsal root ganglion neurons. Eur. J. Neurosci. 28, 1241–1254. 10.1111/j.1460-9568.2008.06438.x 18973552 PMC3111963

[B54] Svaren J. Meijer D. (2008). The molecular machinery of myelin gene transcription in Schwann cells. Glia 56, 1541–1551. 10.1002/glia.20767 18803322 PMC2930200

[B55] Tep C. Kim M. L. Opincariu L. I. Limpert A. S. Chan J. R. Appel B.. (2012). Brain-derived neurotrophic factor (BDNF) induces polarized signaling of small GTPase (Rac1) protein at the onset of Schwann cell myelination through partitioning-defective 3 (Par3) protein. J. Biol. Chem. 287, 1600–1608. 10.1074/jbc.M111.312736 22128191 PMC3256919

[B56] Thiery J. P. Acloque H. Huang R. Y. Nieto M. A. (2009). Epithelial-mesenchymal transitions in development and disease. Cell 139, 871–890. 10.1016/j.cell.2009.11.007 19945376

[B57] Tian J. Cheng H. Wang N. Wang C. (2023). SLERT, as a novel biomarker, orchestrates endometrial cancer metastasis via regulation of BDNF/TRKB signaling. World J. Surg. Oncol. 21, 27. 10.1186/s12957-022-02821-w 36721236 PMC9887878

[B58] Verstappe J. Berx G. (2023). A role for partial epithelial-to-mesenchymal transition in enabling stemness in homeostasis and cancer. Semin. Cancer Biol. 90, 15–28. 10.1016/j.semcancer.2023.02.001 36773819

[B59] Villarreal C. F. Sachs D. Funez M. I. Parada C. A. de Queiroz Cunha F. Ferreira S. H. (2009). The peripheral pro-nociceptive state induced by repetitive inflammatory stimuli involves continuous activation of protein kinase A and protein kinase C epsilon and its Na(V)1.8 sodium channel functional regulation in the primary sensory neuron. Biochem. Pharmacol. 77, 867–877. 10.1016/j.bcp.2008.11.015 19073148

[B60] Wu Q. Hou X. Xia J. Qian X. Miele L. Sarkar F. H.. (2013). Emerging roles of PDGF-D in EMT progression during tumorigenesis. Cancer Treat. Rev. 39, 640–646. 10.1016/j.ctrv.2012.11.006 23261166 PMC3619006

[B61] Xiao J. Wong A. W. Willingham M. M. Kaasinen S. K. Hendry I. A. Howitt J.. (2009). BDNF exerts contrasting effects on peripheral myelination of NGF-dependent and BDNF-dependent DRG neurons. J. Neurosci. 29, 4016–4022. 10.1523/JNEUROSCI.3811-08.2009 19339597 PMC6665359

[B62] Zahalka A. H. Arnal-Estape A. Maryanovich M. Nakahara F. Cruz C. D. Finley L. W. S.. (2017). Adrenergic nerves activate an angio-metabolic switch in prostate cancer. Science 358, 321–326. 10.1126/science.aah5072 29051371 PMC5783182

